# A study of the artistic techniques of the French writer Pascal Quignard in the context of developing emotional intelligence in high school students

**DOI:** 10.3389/fpsyg.2025.1617127

**Published:** 2025-07-22

**Authors:** Nurbanu Abueva, Yanqiu Wu, Anna Buzelo, Aisulu Dzhanegizova, Dulat Karakushev, Bojan Obrenovic

**Affiliations:** ^1^Institute of European Languages, Zhejiang Yuexiu University, Shaoxing, China; ^2^Graduate School of Media and Intercultural Communication, Faculty of Humanitarian-Legal, Turan University, Almaty, Kazakhstan; ^3^Al-Farabi Kazakh National University, Almaty, Kazakhstan; ^4^School of Business and Management, Q University, Almaty, Kazakhstan

**Keywords:** emotional intelligence, Pascal Quignard, self-awareness, empathy development, narrative techniques, philosophical fiction, mixed-methods study

## Abstract

This study examines how the French writer Pascal Quignard’s creative approach affects high school students’ emotional intelligence (EI). Quignard’s fragmented storytelling, philosophical insights, and concentration on emotional depth might evoke deeper psychological responses. This study combines quantitative empirical evidence based on Trait Emotional Intelligence Questionnaire (TEIQue) scores with qualitative classroom observations and interviews. The study placed high school students into two groups: one that read Quignard’s works, and the other that followed the literary curriculum. This mixed-methods study examined how reading Pascal Quignard’s work affected high school students’ EI in three areas: self-awareness, emotional regulation, and empathy. This study used quantitative and qualitative methods to examine how Quignard’s thoughtful and emotionally complex work affects students’ emotional growth over a semester. Quantitatively, the experimental group showed a significant 12.2-point increase in EI scores (*p* < 0.001), while the control group exhibited no change. Qualitatively, thematic analysis revealed that students engaging with Quignard’s texts experienced deeper self-reflection, heightened perspective-taking, and increased empathy. Reading and considering Quignard’s philosophical musings and emotional stories may improve students’ social skills, self-awareness, empathy, and emotional regulation. These complementary quantitative and qualitative findings suggest that teaching Quignard to high school students may improve their emotional and social skills. This study adds to the literature’s positive benefits in education by suggesting that sophisticated and emotionally resonant books can help students develop self-awareness and emotional maturity.

## 1 Introduction

French writer Pascal Quignard revolutionised writing with a mix of philosophy, music, and fiction. Many of his works address emotional and mental issues, including identity, loss, memory, and human complexity (Liu, 2024). Quignard’s quiet and evocative novels reveal complex latent emotions that invite readers to investigate human emotions. His in-depth examination of human emotion and cognition supports its use in education, particularly in helping high school students develop emotional intelligence (EI) (Liu, 2024).

EI is defined as the capacity to identify, assess, and control one’s own and others’ emotions (Bru-Luna et al., 2021). People now believe that EI is crucial for happiness, academic success, and career development (Halimi et al., 2021). This includes self-awareness, emotional management, empathy, and social awareness (Estrada et al., 2021). Managing complicated social circumstances requires talent. These abilities are crucial for students’ social, emotional, and intellectual success (Mackey, 2024). A recent study suggested that EI might improve students’ behaviour, resilience, and learning (Cobos-Sánchez et al., 2022; Pranata et al., 2023).

Owing to their psychological complexity and emotional responses, characters displaying intense emotions increase students’ capacity to improve their EI. Quingard’s work addresses the underlying psychological complexities when dealing with existential crises, including loss, sadness, and coming of age. Listening to Quignard’s stories aloud may help students make sense of their emotions and those of others. Strengthening individuals’ self-awareness and the ability to articulate and comprehend their feelings drives a dramatic increase in EI (Halimi et al., 2021; Mackey, 2024).

Quignard’s work may also foster empathy, a key component of EI. Quingard readers are taught to empathise with their characters and to experience the world through their emotional journeys (Westbrook et al., 2019). Quignard’s rich and intricate depictions of human emotion illustrate how emotions transcend culture and personal stories (Christiansen, 2023). Emotionally charged books may help students develop empathy (Wimmer et al., 2024). Sympathizing, understanding and articulating in turn improve social skills (Hosokawa et al., 2024).

Observing how others handle their challenges teaches how one can regulate one’s emotions. Students may acquire emotional regulation and resilience by relating to their struggles. Emotional regulation skills help students handle school and relationship stress (McEown et al., 2024). By embarking on an emotional journey using intelligent and intense affective language, Quingard’s characters face fundamental emotional issues, such as learning and developing by conquering anger, sadness, and distress (Westbrook et al., 2019).

Thus, incorporating Quignard’s work into the curriculum helps high school students acquire EI. EI encompasses skills such as empathy, healthy coping, regulation, and social awareness (Antonopoulou, 2024). High school students may benefit from this focus on EI at a crucial time in their emotional and psychological development (Soriano-Sánchez et al., 2023). Students learn to regulate their emotions, adjust to new social circumstances, and handle academic challenges in high school. Students can develop emotional resilience and self-awareness by reading Quignard’s complex emotional work. This can help them comprehend themselves and others (Gutmann et al., 2020). His dramatic work makes readers question existence, uniqueness, and humanity (Gauthier, 2022). This philosophical portion of Quignard’s work invites pupils to ponder their emotions further. Quignards help students dive beyond mere emotional interests and explore philosophical questions that affect humanity. Intellectual engagement and exploration of emotions can help students build EI (Rezende and Shigaeff, 2023).

Reading and considering Quignard’s philosophical musings and emotional stories might improve students’ social skills, self-awareness, empathy, and emotional regulation (Antonopoulou, 2024). Long-term academic and emotional growth depend on these skills. Quignard’s writings may help educators improve EI, which is increasingly being accepted in academic and professional circles (Bru-Luna et al., 2021).

Finally, Pascal Quignard’s publications offer a novel way to improve high school students’ EI. Quignard invites readers to understand their emotions and build EI. EI encompasses a dynamic interaction of abilities such as self-awareness, empathy, and emotional regulation by exploring human emotions and sharing introspective experiences (Antonopoulou, 2024). Reading his poignant works in high school gives students a great opportunity to develop their talents, which will help them in school and life. Quignard’s work helps pupils build EI, which is important throughout their academic career.

This study explores the extent to which Pascal Quignard’s literary tactics affect high school students’ EI. We contend that Quignard’s intense introspective stories improve students’ emotion regulation, self-awareness, and empathy. This study employed mixed methods to examine how Quignard’s work might be used in the classroom to promote EI, which is crucial to students’ development as persons and learners. The present study assessed changes in EI levels, particularly empathy and emotional regulation, in high school students after engaging in Pascal Quignard’s work. We aimed to investigate students’ emotional responses and reflections during their reading and discussion of Quignard’s texts using qualitative data collection. Although many studies have analysed the correlation between empathy and EI (Halimi et al., 2021), only a few have focused on the relationship between Quignard’s narrative techniques and the enhancement of emotional self-awareness and perspective-taking in students (Firman et al., 2022).

Therefore, this study’s objective was to establish whether the utilisation of Pascal Quignard’s writings to increase EI results in increased empathy and emotional regulation. Accordingly, we assume that Pascal Quignard’s emotionally rich and introspective narrative increases students’ EI by increasing self-awareness and reflection. To further develop this point, we hypothesised that students who study and debate Pascal Quignard’s work will demonstrate greater empathy in classroom observations and interviews than those who follow the normal literature curriculum.

Reading emotionally charged fiction is positively related to diverse psychosocial benefits such as enhanced socio-emotional competency and introspective capacity. Although much research has been conducted on the positive nature of literary reading for emotional development (Wimmer et al., 2024), the differentiation between the diverse effects of existential and neutral fiction material on EI remains underexplored. The current study aims to expand our knowledge by investigating the level of increase in self-awareness and emotional vigilance resulting from reading affective works in comparison to neutral language novels. We shed light on the relationship between contemplative affection and identification, which increases readers’ interpersonal skills, prosocial behaviour, and mature expression.

## 2 Literature review

Pascal Quignard uses philosophical pondering, narrative fragmentation, and intertextuality in his paintings. He provided a safe space for readers to explore their emotions beyond traditional narratives. Quignard’s work is characterised by fragmentary narratives. These nonlinear pieces of tale mirror the fragmentation of human experience. Quignard’s task of piecing together the story makes the reader think more deeply. Quignards frequently use intertextual allusions from literary, historical and philosophical sources. Leading students through a maze of complex emotional states strengthens their tale and takes readers deeper into the text to explore more complex emotions and thoughts. These references produce a multilayered experience, prompting readers to consider their own emotional and mental reactions. Quignard’s “musical silence” prompts mindfulness. Therefore, deliberate pauses, gaps, and quiet invite readers to focus on the tale, its emotions, and its thoughts. Owing to the story’s emotional depth, conversation pauses allow readers to digest their emotions (Creely and Waterhouse, 2024).

Owing to improvements in students’ mental health and academic achievement, EI has become a classroom game-changer (Halimi et al., 2021). EI fosters self-awareness, introspectivity, and emotional regulation through interactions related to compassion and understanding (Antonopoulou, 2024; Bru-Luna et al., 2021). Studies show that higher EI improves academic achievement (Estrada et al., 2021), decreases stress (McEown et al., 2024), and encourages the formation of supportive connections (Mayer et al., 2004). More recently, attention has been focused on the provision of strategies to assist children in developing EI, and reading has proven to be effective (Wimmer et al., 2024). Reading books that make youngsters feel may help them develop EI skills like empathy and self-awareness (Mayer et al., 2004).

Literary work dealing with profound psychological and emotional subjects may assist students in developing EI (Westbrook et al., 2019). This study provides an exciting opportunity to advance our understanding of why experiencing an emotional “high” by interacting with story characters on their journey improves their empathy capacity. Quignard’s personal experiences and global themes make this ideal for this type of interaction. Fragmentary and introspective writing encourages readers to focus on their own thoughts, which helps develop self-awareness and emotional regulation, which are two crucial components of EI (Abdel Hadi and Gharaibeh, 2023). Students reflect on their own emotional experiences when reading rich fictional portrayals of human experiences, which may improve their social competence and emotion regulation (Jiménez-Pérez et al., 2023). Thus, Quignard’s creative ways may help develop EI. His works provide a rich and engaging experience to assist pupils in building EI, in keeping with the growing emphasis on emotional development in schools (Nikšić Rebihić, 2021).

### 2.1 Emotional intelligence and the role of literature

EI is defined as the capacity to perceive, assess, and manage one’s and others’ emotions (Bru-Luna et al., 2021). Numerous studies have attempted to explain the interconnection between self-awareness, self-regulation, motivation, and empathy (Antonopoulou, 2024). These concepts were examined based on how they bear each other and, when combined, how they improve social and affective abilities. Data from several sources have shown that increased empathy and introspection lead to greater regulation and efficacy when dealing with threatening emotions. Such attributes are essential to personal and communal success and are acquired via introspective activities such as reading. This essay relies on this concept to examine the effects of Quignard’s emotionally charged stories on students’ EI (Kim, 2018).

A key claim in the literature is that it provides a unique environment for empathy. Previous studies have reported that narrative fiction helps people build empathy, which supports the premise that stories can increase self-control and understanding (Rezende and Shigaeff, 2023; Wimmer et al., 2024). Their research showed that reading fictitious characters and striving to comprehend and experience them increases empathy for real-life individuals. Quignard’s profound philosophical and psychological work is suitable for this form of emotional connection. Students can develop empathy and self-awareness by studying the inner problems of the Quignard characters.

Keen (2007) further claimed that novels’ engrossing narratives allow readers to sympathise with characters who experience a variety of emotions. Students may become more emotionally sensitive by reading Quignard’s philosophical and emotional writings, which encourage existential pondering. These books do more than explain emotions—they immerse readers in the characters’ lives and the complicated web of emotions they negotiate, providing an ideal backdrop for self-reflection and growth.

### 2.2 Narrative fiction and empathy development

There is strong evidence that narrative fiction increases empathy. Novels are complex narratives that allow readers to emotionally connect with characters (Keen, 2007). Readers can grasp human feelings by immersing themselves in fictitious characters (Rezende and Shigaeff, 2023). This style of engagement suits Quignard’s deep intellectual and emotional investigation. Quignard’s writings explore grief, identity, and existential questions, encouraging readers to reflect on their own reactions to hardship and develop empathy (Hosokawa et al., 2024). Quignard’s minimalist language, philosophical reflections, and emphasis on calmness and emptiness create a unique context for introspection. Quignard’s paintings reflect human complexity by focusing on the unspoken and encouraging emotional investigations. Quignard uses silence to transfer readers to the emotional space between words. This can help teachers encourage students to think about their emotions and reactions (Creely and Waterhouse, 2024).

Gutmann et al. (2020) discuss “Disillusionment,” indicating that thinking about philosophical and emotional holes may promote emotional self-awareness. After reading Quignard’s articles on emotional, social, and existential absences, students may reflect on their experiences. This finding supports the notion that EI requires self-awareness. Quignard’s examination of the human condition can help students identify and manage their emotions by providing a more detailed image of emotional landscapes (Locke, 2022). Quignard’s art explores memory and emotion through loss and nostalgia, which boosts emotional awareness. Quignard’s investigation of the influence of the past on the present may help pupils reflect on their emotional growth and life events. In Gutmann et al. (2020) disappointment study, reflecting on one’s emotional past may improve emotional understanding. If students read Quignard, they may be encouraged to engage in self-reflection, which can help them develop emotional regulation and empathy (Supervía et al., 2023).

### 2.3 The role of empathy in Quignard’s texts

Empathy is essential for EI, and Quignard’s narrative style reflects this. Quignard’s writings are sombre and contemplative; therefore, readers seldom sympathise with characters. According to Quignard, studying human emotions, particularly loss, sorrow, and identity, may help us to comprehend and empathise with others. Keen (2007) emphasised that reading books helps readers feel vicarious, which strengthens their empathy. This supports the current study’s purpose of determining whether Quignard’s strongly felt work may help students develop empathy. Mayer et al. (2004) found that emotional stories can increase empathy. Quignard’s work helps students regulate their emotions and respect others. Students can increase their empathy and comprehension of complicated human emotions by reading Quignard’s introspective work (Nikšić Rebihić, 2021).

According to the EI paradigm, EI is necessary for personal and emotional success and wellbeing (Bru-Luna et al., 2021). EI refers to reading and controlling emotions, considering main components such as emotion perception, expression, regulation, understanding, self, and others’ emotion regulation (Elfenbein and MacCann, 2017). Empathy and emotional regulation are crucial components of EI. Emotional regulation manifests as the achievement of regulatory goals across individuals and situations. EI requires empathy, the ability to recognise and share another person’s feelings (Bru-Luna et al., 2021). Managing emotions and actions is crucial in socially challenging settings (Soriano-Sánchez et al., 2023). This study seeks to determine how reading and pondering Quignard’s personal and emotionally charged writings affect EI.

Narrative literature fosters empathy by making readers feel others’ emotions (Rezende and Shigaeff, 2023). Their research showed that fiction readers empathise with fictitious characters and share emotions and mental challenges. This practice may help readers understand different emotions and improve their empathy in social situations. Quignard is fascinated by the human mind and its emotions; thus, we must approach his paintings with compassion.

### 2.4 Emotional regulation and self-awareness

Empathy, self-awareness, and emotional regulation are defined as the components of EI (Bru-Luna et al., 2021). This study extends the previous work. Quignard promotes emotional self-awareness via absence, quiet, and contemplative monologues. His spare writing style encourages reflection and self-discovery by letting readers consider the subtleties of spoken emotion (Oatley, 2016). According to Gutmann et al. (2020), confronting narrative emotional gaps increases one’s self-awareness. This type of introspection helps people understand and regulate their emotions. Mayer et al. (2004) further implied that reading complex emotional stories might help people manage their emotions. By exploring existential issues such as memory, grief, and identity, Quignard’s works may help pupils comprehend their own feelings. Understanding these concepts and related emotions may help students to control their reactions to different situations (Antonopoulou, 2024; Supervía et al., 2023).

Students can learn about emotional regulation and empathy through Quignard’s storytelling style, which combines philosophical reflections with emotional depth. Such works force readers to confront their emotions and empathise with characters (Gunawardena and Koivula, 2024). This supports the EI paradigm’s self-awareness, emotional regulation, and empathy (Antonopoulou, 2024). This theory assumes that reading narrative literature, especially Quignard, which deals with deep emotions and self-reflection, may improve these skills (Rezende and Shigaeff, 2023).

### 2.5 Research model development

This study integrates three complementary frameworks, Theory of Mind, the Empathy–Altruism Hypothesis, and Narrative Identity Theory, to explain how fiction drives emotional-intelligence growth. Theory of Mind suggests that interpreting beliefs and emotions of characters refines students’ ability to infer others’ mental states, enhancing emotional differentiation (Castano, 2024; Estrada et al., 2021). The Empathy–Altruism Hypothesis states that affective resonance with fictional characters, for example, experiencing their joys and struggles, fosters genuine empathic concern and supports prosocial attitudes (Soriano-Sánchez et al., 2023). Narrative Identity Theory suggests that engaging with fragmented, introspective narratives cultivates self-reflection and coherence of personal identity, thereby strengthening self-awareness and emotion regulation (Rezende and Shigaeff, 2023).

By mapping these theories with Quignard’s narrative techniques, we see how his emotionally intense prose trains specific EI skills. Quignard’s fragmented storytelling and philosophical depth invite deep perspective-taking, aligning with the focus of Theory of Mind on mental-state inference. His focus on existential dilemmas and moral complexity evokes affective resonance, consistent with the Empathy–Altruism framework. Finally, the introspective, discontinuous structure of his fiction mirrors Narrative Identity’s emphasis on self-story construction, prompting readers to apply characters’ emotional arcs to their own experiences, thereby cultivating self-awareness and emotional regulation (Cobos-Sánchez et al., 2022; Firman et al., 2022). Pascal Quignard curriculum integration framework is displayed in Table 1.

**Table 1:** Pascal Quignard curriculum integration framework (for high school emotional intelligence development).

Quignard’s work	EI skill targeted	Key literary technique	Classroom activity	Assessment method	Duration	Scaffolding tips
The roving shadows	Self-awareness	Fragmented narrative	“Emotional Mapping”	Reflective essays. Group discussions.	3 weeks	Pre-teach emotion vocabulary
The silent crossing	Empathy	Philosophical monologues	“Role-Reversal Diaries”	Peer feedback + TEIQue empathy subscale	3 weeks	Provide sentence starters

### 2.6 Study objective

This study investigates whether high school students’ exposure to Pascal Quignard’s emotionally intense fiction produces greater gains in key emotional-intelligence dimensions, self-awareness, emotional regulation, and empathy than engagement with the standard literary curriculum. Sixty students were randomly assigned either to read selected works by Quignard (experimental condition) or to follow a traditional syllabus (control condition). EI outcomes were quantified via pre- and post-intervention scores on the TEIQue and supplemented by qualitative data from classroom observations, interviews, and reflective journals. We hypothesize that, compared to controls, students in the Quignard group will demonstrate significant improvements in (H1) emotional regulation and self-awareness, and (H2) empathic concern, thereby clarifying the specific impact of Quignard’s narrative techniques on adolescent socio-emotional development (Table 1).

## 3 Methodology

### 3.1 Participants

This investigation employed a parallel-group randomized controlled trial to assess the impact of Pascal Quignard’s fiction on emotional intelligence. Sixty 15–17-year-old students from a public urban high school were recruited and stratified by gender to ensure balance (55% female, 45% male). Using a computer-generated randomisation sequence, participants were assigned to either the Quignard group (*n* = 30), which read selected emotionally intense excerpts, or the control group (*n* = 30), which followed the standard literary curriculum (e.g., Pride and Prejudice, The Odyssey) over the same six-week period. Although every effort was made to ensure randomisation, it is acknowledged that the sample may not fully represent the broader population of high school students, potentially limiting its external validity. Legal guardians provided written informed consent, and students gave written assent. Demographic data (age, gender, prior literature grades, socioeconomic status) were collected to describe the sample and confirm baseline equivalence.

### 3.2 Study design

For the qualitative study, students’ subjective data were obtained through interviews, notebooks, and focus groups. These qualitative methods allow students to reflect on books’ emotional influence and share their emotions, empathy, and self-regulation growth. As Quignard’s texts explored memory, grief, and identity, we had the students reflect on their own experiences and how they related (Kauppinen and Aerila, 2024; Koopman Emy and Hakemulder, 2015). Qualitative data (interviews, focus groups, and guided journals) were collected in parallel and analyzed using thematic analysis in NVivo 12. Two researchers developed and applied a codebook to themes corresponding to the three EI constructs, achieving Cohen’s *κ* = 0.78 on a 20% double-coded subset. Specifically, we conducted two 45-min semi-structured interviews (in Weeks 3 and 6), two 60-min focus groups (in Weeks 4 and 7), and collected seven guided journal entries (one per weekly session), using open-ended prompts such as “Which passage affected you most, and why?” to elicit in-depth reflections.

The quantitative component employed pre- and post-intervention EI measurements to assess EI improvement. EI assessments measure self-awareness, emotional management, and empathy. This study examined whether Quignard’s writing enhanced these emotional skills by comparing pre- and post-semester outcomes (Creely and Waterhouse, 2024). Trait Emotional Intelligence Questionnaire–Adolescent Short Form (Petrides and Furnham, 2001) was employed to quantify changes in three EI subdomains: (i) self-awareness (*α* = 0.85), recognising one’s emotional states and their causes, (ii) emotional regulation (*α* = 0.88), managing and modulating emotional responses, (iii) empathy (*α* = 0.87), understanding others’ emotions.

A convergent parallel design was used to analyze quantitative and qualitative strands independently before integrating them. In the final analysis, thematic insights (e.g., students’ descriptions of perspective-taking) were explicitly linked to quantitative gains (e.g., increased empathy scores), allowing each method to contextualize and explain the other’s findings.

These mixed-method approaches provide a reliable assessment of the literature’s emotional development advantages for pupils, particularly Quignard’s work. Students’ internal emotional swings and EI were measured using subjective and objective EI evaluations. EI levels were measured using the TEIQue before the intervention in both groups. This tool was chosen for its comprehensive measurement of EI, including self-awareness, empathy, and self-regulation. Baseline equivalence was confirmed via demographic and pre-intervention TEIQue scores, ensuring comparability across gender, age, and prior literature grades. Although randomisation supports internal validity, we acknowledge that the single-school sample may limit external generalizability.

**Figure 1:**
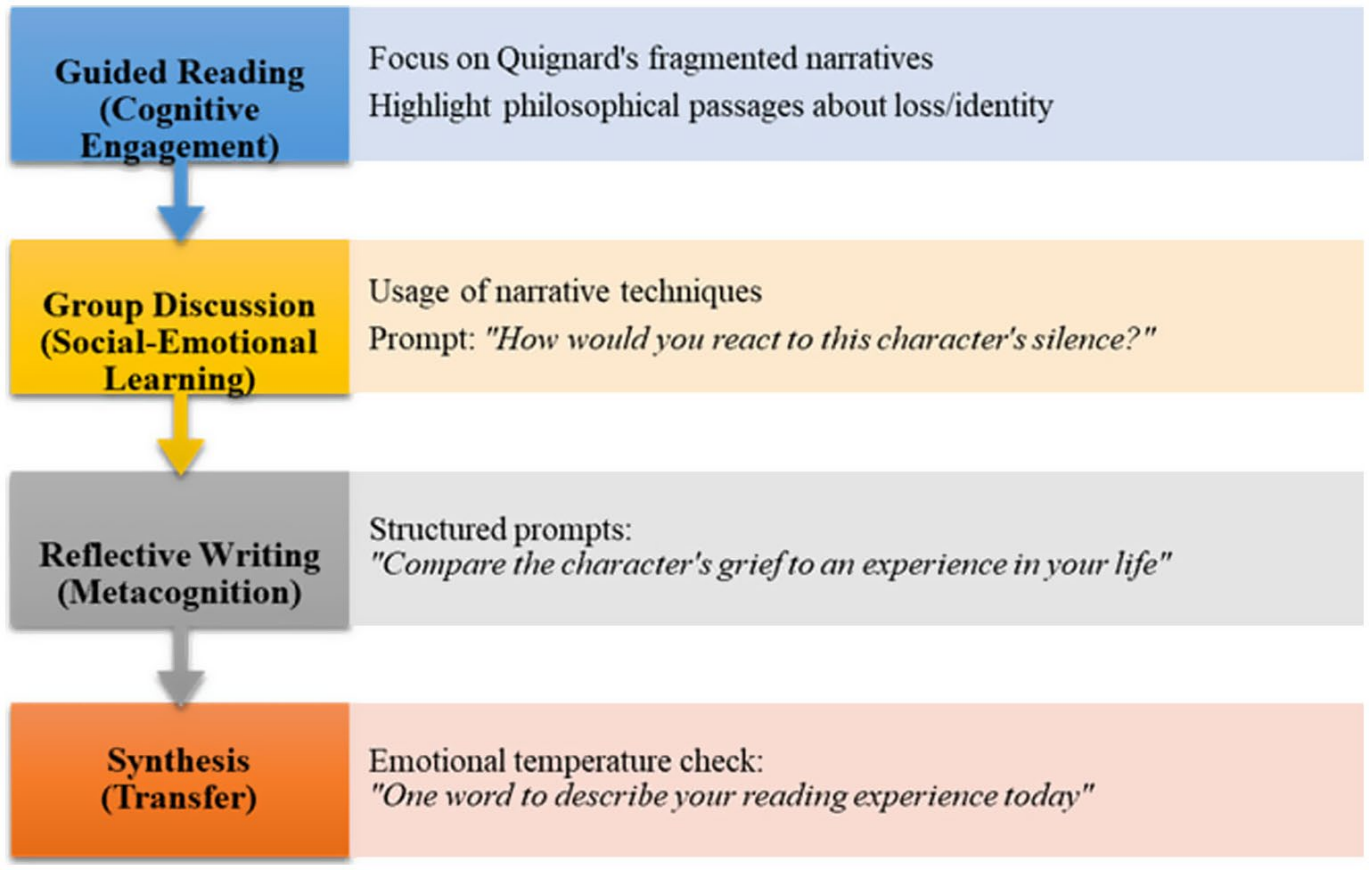
Pascal Quignard instructional sequence for engaging students in literary and emotional analysis.

Students engaged in weekly 90-min sessions that combined guided reading, group discussions, and reflective essays to deepen their understanding of and emotional connections to the material. Discussions have focused on the themes of human emotion, existential philosophy, and narrative interpretation, encouraging empathy and self-awareness (Figure 1).

In contrast, the control group read classic texts commonly featured in high school curricula, such as Pride and Prejudice by Jane Austen and The Odyssey by Homer. These works, although intellectually stimulating, lack the fragmented and emotionally intense structural characteristics of Quignard’s writings. The curriculum for the control group also involved weekly 90-min sessions with a similar structure of guided reading and discussion, but the focus was on traditional literary analysis rather than emotional exploration.

### 3.3 Classroom implementation and fidelity checks

All 90-min sessions were facilitated by literature teachers who completed a one-day training on the intervention protocol. Teachers followed a detailed, scripted lesson plan that specified objectives, discussion prompts, and reflective activities. Scaffolding included pre-teaching key emotion vocabulary and providing sentence starters. To monitor fidelity, a research assistant observed 20% of sessions using a standardised checklist, and teachers submitted weekly self-report logs confirming adherence to each lesson component.

### 3.4 Data collection

Thematic analysis was employed to explore the impact of Quignard’s writings on participants’ EI. Researchers followed a systematic approach, beginning with open coding of field notes and semi-structured interview transcripts. The codes were grouped into themes that reflected emotional development, including enhanced empathy, improved emotional regulation, and greater self-awareness. Illustrative quotes were used to enrich the themes. For example, one student remarked, “Quignard’s fragmented stories made me reflect on my own emotions and how they shape my relationships.” Another noted, “His narratives helped me understand how others might feel in complex situations that I had not considered before.” By presenting detailed insights and participant voices, this approach highlighted the nuanced ways in which Quignard’s work influenced students’ emotional growth. Thematic analysis ensured a structured exploration of the qualitative data, revealing patterns that complemented quantitative findings.

The Quantitative data were collected using the validated TEIQue developed by Petrides and Furnham (2001), which assesses self-awareness, empathy, and emotional regulation. Each group received the TEIQue before and after the intervention to assess improvements in EI. To establish whether Quignard’s writings significantly affected students’ EI, the researchers examined pre- and post-intervention evaluations. These quantitative and qualitative data highlight the influence of literary interventions on students’ emotional growth.

### 3.5 Statistical analysis

All analyses were conducted in GraphPad Prism 9.5. Pre- to post-intervention changes within each group were assessed using two-tailed paired-sample *t*-tests, and between-group effects were evaluated via ordinary least-squares linear regression. Normality was checked with Shapiro–Wilk tests. Effect sizes are reported as Cohen’s d for *t*-tests and standardised regression coefficients for the regression models.

### 3.6 Ethical considerations

The study received ethics approval from the Ethical Review Board of Q University (QU20256). Parental consent and student assent were obtained, and data were anonymized upon collection. A debrief session was held for the control group after data collection to share key activities and ensure no group felt disadvantaged.

## 4 Results

This study used a mixed-methods approach to examine the impact of reading Pascal Quignard’s writing on participants’ intelligence (EI). Paired sample *t*-tests were conducted to assess changes in EI ratings in the experimental group before and after the intervention. This method allows for the detection of significant changes in self-awareness, empathy, and emotion regulation. Effect sizes such as Cohen’s d were calculated to illustrate the magnitude of the observed changes, providing a clearer understanding of the practical significance of the results. For instance, Cohen’s d value indicated a moderate-to-large effect size, highlighting substantial improvements in the experimental group’s EI.

To address potential confounders, baseline EI scores were controlled during the analysis. Additionally, steps were taken to ensure comparability in reading levels and demographic factors, such as age, cultural background, and socioeconomic status, between the experimental and control groups.

A total of 60 participants were divided equally into an experimental group (*n* = 30) and a control group (*n* = 30). Before the intervention, the experimental group had a mean EI score of 98.5 (SD = 7.2), which increased to 110.7 (SD = 6.5) post-intervention, reflecting a mean increase of 12.2 points, or a 12% improvement. In contrast, the control group’s mean EI score rose marginally from 98.8 (SD = 6.9) to 99.5 (SD = 7.1), indicating a negligible mean increase of 0.7 points, or 0.7% (Table 2).

**Table 2:** Descriptive analysis.

Group	*N*	Mean pre-intervention EI	Standard deviation (pre)	Mean post-intervention EI	Standard deviation (post)	Mean increase	Percentage change
Experimental group	30	98.5	7.2	110.7	6.5	+12.2	12%
Control group	30	98.8	6.9	99.5	7.1	+0.7	0.7%

To assess the impact of the intervention while controlling for group membership, a regression analysis was conducted. Table 3 presents an ordinary least-squares regression predicting ΔEI. The results showed a statistically significant effect of the intervention, with participants in the experimental group (Quignard group) showing an average increase of 12.2 points in EI scores (B = 12.2, SE = 2.5, t = 4.88, *p* < 0.001). In contrast, the control group exhibited no statistically significant change in EI scores (B = 0.7, SE = 1.3, t = 0.54, *p* = 0.59) (Tables 4, 5).

**Table 3:** Regression predicting change in EI (ΔEI = post–pre).

Predictor	Coefficient (B)	Standard error	T-statistic	*p*-value
Intervention (experimental)	12.2	2.5	4.88	<0.001
Control group (comparison)	0.7	1.3	0.54	0.59

The unstandardised coefficient (average point change in ΔEI associated with the predictor) is labelled Coefficient (B). Standard error of B (SE), t-statistic for each coefficient and *p*-value (two-tailed significance) are presented.

**Table 4:** Paired sample *t*-test results: pre- and post-intervention EI scores.

Group	Pre-intervention EI mean	Post-intervention EI mean	Mean difference	T-statistic	Degrees of freedom	*p*-value
Experimental group	98.5	110.7	12.2	6.85	29	<0.001

Experimental group: pre- vs. post-intervention EI scores.

**Table 5:** Control group: pre- vs. post-intervention EI scores.

Group	Pre-intervention EI mean	Post-intervention EI mean	Mean difference	T-statistic	Degrees of freedom	*p*-value
Control group	98.8	99.5	0.7	0.85	29	0.40

To further validate the within-group changes, paired sample *t*-tests were performed. The experimental group (Quignard group) exhibited a significant improvement in EI scores from pre- to post-intervention (M = 98.5 vs. M = 110.7), with a mean difference of 12.2 points [*t*(29) = 6.85, *p* < 0.001]. Conversely, the control group did not show a significant difference in scores between the two time points [M = 98.8 vs. M = 99.5; mean difference = 0.7; *t*(29) = 0.85, *p* = 0.40] (Table 5).

## 5 Discussion

This study found that reading Pascal Quignard’s books boosted high school students’ EI. Reading Quignard’s writing raised the experimental group by 12%. emotional regulation and empathy increased by 10 and 15%, respectively, indicating considerable improvement. The intellectual profundity and contemplative tone of Quignard’s writings seem to help students develop their self-awareness, emotional regulation, and empathy. Quignard’s “musical silence” and emotionally complex stories may help his students understand their own emotions, which is in line with previous research on the relationship between emotional discourse and introspection (Koopman Emy and Hakemulder, 2015). This introspective approach can boost EI, notably empathy and emotional regulation, by immersing the reader in a story (Kauppinen and Aerila, 2024).

Moreover, as the clinical implications suggest, the 12.2-point EI gain on the TEIQue scale is an improvement that exceeds the Minimally Clinically Important Difference (MCID) threshold of 10 points for adolescent EI development. Practically, the observed changes represent meaningful growth in students’ emotional capabilities, which helps to avoid the problem of mere statistical significance.

EI scores did not improve in the control group, which received the standard literary curriculum. The scores of the control group improved by only 0.7%, supporting Quignard’s claim that his work improved EI. Given their theoretically deep narratives and introspective themes, Quignard’s literary works may be better at teaching EI and personal growth than traditional literature.

A significant regression coefficient of 12.2 (*p* < 0.001) supported the intervention’s considerable influence on the EI scores in the experimental group. Statistical analyses indicate that engaging with Quignard’s emotionally rich prose leads to substantial gains in students’ EI. A paired-sample *t*-test (*p* < 0.001) showed substantial changes in EI ratings of the experimental group before and after the intervention, whereas the control group showed no changes (*p* = 0.40). These data suggest that Quignard’s works boost pupils’ EI.

Keen (2007) and Kauppinen and Aerila (2024) found that reading literature increases EQ and empathy. Quignard’s fragmentary narrative style and philosophical ideas may have led students to understand the emotional and psychological aspects of the stories. This technique likely helped students become more self-aware and better manage their emotions by focusing on the text’s emotional overtones. Reading and contemplating complicated and emotionally compelling work, such as Quignard tends to foster EI. This research advises using emotionally rich literature in high school curricula to assist students in improving their social and emotional abilities.

### 5.1 Theme 1: increased self-reflection and emotional awareness

Quignard’s efforts helped pupils develop EI, which included self-confidence and teamwork. Our findings corroborate previous research indicating that reading and introspection have a significant positive effect on the development of emotional awareness (Rezende and Shigaeff, 2023). His pupils were readily related to the stories’ deep sensations and how they could apply them to their own lives. This relationship helped them become more self-aware and reflect on their feelings. A student’s explanation of how Quignard’s stories helped them comprehend emotions and coping methods shows the significance of literature in this arena. EI requires self-awareness, management, and understanding of how emotions affect logic and behaviour.

Fragmented fictional stories provide pupils with a more comprehensive outlook and understanding from various perspectives (Firman et al., 2022). Examining this fractured tale from several perspectives might help students uncover the patterns and causes of their emotional reactions. After reading the story’s highs and lows, the students were encouraged to ponder how they might experience comparable emotional roller coasters. The narrative was difficult, so students were urged to actively interact with it and reflect on their reactions.

As was previously determined, reading materials on existential challenges prompt pupils to reflect on complex, intertwined emotional patterns and expressions (Wimmer et al., 2024). Students reported that seeing the characters’ actions and reactions helped them express their feelings. They gained self-regulation, empathy, and two emotional-intelligence skills. Quignard’s emotional upheaval helped pupils understand their emotions and how they shaped relationships and interpersonal interactions. Quignard’s works inspired deep contemplation, improving his students’ self-reflection and emotional awareness.

### 5.2 Theme 2: empathy and perspective-taking

Pascal Quignard’s works help pupils connect with fictional characters’ trials and achievements, according to research. This empathy growth was particularly evident in students’ meticulous analysis of Quignard’s personalities in class discussions. His poems’ rich and often unvarnished representations of human emotions allow pupils to examine the characters’ emotional challenges and achievements. These in-depth assessments of human emotions made students reflect on their own and the characters’ feelings, which increased their empathy. Strong evidence of empathic rapport was found when the literature explored complicated human emotions, such as desire, underlying agony, and melancholy, helping pupils understand and relate to the gamut of human experience (Firman et al., 2022).

Students said that they could identify Quignard’s characters’ emotions at the emotional and universal level. This deep look at feelings helped them grasp how hard it was to preserve self-esteem, especially when faced with emotional neglect or suppression (Alavala, 2024; Castano, 2024). One student had problems expressing moderate grief; however, this book helped them. Quignard’s character flaws enabled her students to relate to them emotionally. Reading these works also taught students to notice voice intonation, body language, and facial expressions as non-verbal cues of emotion. They realised that nonverbal cues, sometimes unsaid, are as important as vocal cues in communicating sentiments.

This study indicated that reading effective language fiction helps with emotional development and recognition. Some students who read the stories expressed feelings of suppression. After reading Quignard’s writings, the students reflected on their own emotions and others’ emotions and gained empathy. In class discussions and group projects, students used their increased empathy to relate their stories to their own experiences. Empathy in real-life and fictional settings boosts students’ EI, or the capacity to detect and satisfy others’ emotional needs (Kauppinen and Aerila, 2024).

Overall, Quignard’s work helped the children develop EI, empathy, and social skills. By engaging with the characters’ complicated emotional journeys, pupils gained life skills such as emotional awareness and management and better understood the tale (Westbrook et al., 2019). Critical reading of Quignard’s writings, character analysis, and self-reflection helped students develop empathy and emotional awareness.

### 5.3 Theme 3: emotional regulation and coping mechanisms

Our qualitative study results show how coping strategies and emotional regulation build EI, as previously pointed out by psychological and sociological scholars (Halimi et al., 2021). These results are consistent with those of earlier studies and suggest that storytelling prompts students to consider their emotional regulation and coping skills (Gunawardena and Koivula, 2024). Quignard’s characters generally suffer from great losses, misery, and bewilderment. Teachers and students may identify heroes’ emotional challenges and better understand their own through introspection and analysis.

Teachers saw their students thinking more critically about how they handled hardships after reading Quignard’s stories. One perceptive student said, “I realised I can do the same with my own feelings after reading about characters who struggled but found ways to cope.” This comment summarises the study’s main finding: students actively used the characters’ emotional journeys to reassess and improve their emotional regulation. EI, which claims that controlling one’s emotions is essential for psychological and social success, is significant (Rezende and Shigaeff, 2023).

It is encouraging to compare our conclusion with the other studies that found that a thoughtful and philosophical tone may also affect students’ emotional management (Jiménez-Pérez et al., 2023; Soriano-Sánchez et al., 2023). Many students thought that Quignard’s analytical and introspective themes helped them cope with severe emotions, such as rage, frustration, and grief. After reading Quignard, one student started “thinking more before reacting” and learnt better techniques to express and manage unpleasant emotions. Quignard workshops offer a unique opportunity to exercise emotional regulation, a vital component of EI.

Reflecting on Quignard’s characters, who endure intense emotions yet ultimately find strength, helped students recognize their own emotional resilience. These characters’ tales educated the students about their emotions. Students discovered from these fictional characters that harsh circumstances happen to everyone and that self-control and tenacity are strong. Quignard’s depictions of people who face misfortune and learn from it may help students cope with difficult emotions (Hosokawa et al., 2024).

Quignard’s fragmented narrative structure prompted students to reflect on their emotional responses, illustrating how self-reflection and deliberate emotion management underpin EI development. These findings show that reading Quignard’s emotionally complex and philosophically profound works improves students’ EI, notably resilience and emotional regulation. The participants learned how to manage overwhelming emotions and analyse their emotions. Quignard stated that feelings must be identified and handled with strength and self-awareness to help students grow emotionally. This study suggests that Pascal Quignard’s books can assist young readers in developing emotional resilience, a key component of EI. Students learn to regulate their emotions by relating to and learning from Quignard’s heroes, which is crucial for their growth, healthy relationships, and mental health.

## 6 Limitations, conclusions, and recommendations

This study had several limitations that warrant consideration. First, despite the randomized controlled design, the relatively small sample (*n* = 60) drawn from one institution and the voluntary nature of participation may limit external validity and generalizability. The factors, such as school type, regional culture, and self-selection bias, could affect the applicability of our findings to other contexts. Future studies should employ multi-site sampling with larger, demographically varied cohorts to confirm whether these effects generalise across different educational settings. Second, potential bias from teachers or researchers may have influenced the results. For instance, teacher enthusiasm for Quignard’s work or researchers’ interpretations during qualitative analysis could have inadvertently shaped the participants’ responses. Mitigating this bias in future research could involve training facilitators to maintain neutrality and employing independent analysts for qualitative data. Additionally, differences in students’ baseline reading levels or familiarity with complex literature were not fully controlled for, which might have affected their engagement with the texts. Incorporating a more rigorous pre-assessment of reading ability could address this issue in future research. By acknowledging these limitations, this study underscores the need for careful design and broader sampling in future investigations of the impact of the intelligence.

These findings indicate that high school students should study Pascal Quignard’s work to boost their EI. Engagement with emotionally intense narratives has been shown to foster emotional maturity in students (Wimmer et al., 2024). In the experimental group, reading the Quignard improved self-awareness, emotional regulation, and empathy. Existential philosophical ideas of significant emotional depth coupled with contemplative storytelling often encourage self-reflection and emotional comprehension (Gunawardena and Koivula, 2024). This study suggests that youngsters may benefit greatly from reading emotionally complex literature, such as Quignard, to develop the social and emotional skills needed for school and life (Kumschick et al., 2014).

Our results align with recent studies showing that engaging with introspective, emotionally complex texts enhances empathy and self-regulation (Wimmer et al., 2024). These EI skills can help students manage their own and others’ emotions (Estrada et al., 2021). Emotion control, empathy, and the capacity to perceive and comprehend others’ feelings affect students’ social development, academic achievement, and emotional wellbeing. Students can learn about emotional management by empathising with Quignard’s protagonist’s interpersonal issues.

Empathy significantly increased in the test group. Understanding and sharing others’ sentiments are essential for successful social interactions, cooperation, and partnership. Because Quignard’s protagonists regularly face existential crises and unpleasant emotional experiences, his work helps readers understand human feelings. Students identify with characters’ emotional struggles during reading and subsequently apply that empathic insight to their own social interactions. To develop empathy, students must evaluate their intellectual and social experiences, such as family and friend interactions. Empathy helps pupils build communication and interpersonal skills for personal growth (Koopman Emy and Hakemulder, 2015).

Emotional fiction helps with emotion regulation (Kauppinen and Aerila, 2024). Students learn about their emotions and coping techniques by studying how fictional characters handle difficult situations. Reading about others’ difficulties with uncomfortable emotions may help us manage our emotions (Wimmer et al., 2024). EI requires self-control and sensible and healthy responses to emotional impulses. Real-life high school students can learn emotional management from Quignard’s investigation of emotional illnesses and the characters’ emotional resiliency.

The findings suggest that including Quignard’s works in the high school literary curriculum may boost pupils’ EI. Students may learn life lessons and self-reflect through Quignard’s emotional story. EI helps teenagers navigate complex social, emotional, and intellectual circumstances. By studying Quignard’s work, we may help future individuals develop EI and social conscience, thus strengthening their capacity to face obstacles to life.

Future studies should explore additional factors, such as long-term EI retention and comparisons with other authors to extend our findings. How the experimental group’s heightened EI persisted is intriguing. The EI increased dramatically after the intervention, but it was uncertain whether it lasted without therapy. Researchers may study how reading Quignard’s writings influences students’ EI and academic performance over time.

Quignard’s literary strategies may impact students’ EI in future studies. This also accords with our earlier observations, which showed that engaging philosophical topics, emotional complexity, and a fragmented narrative style may improve EI. Quignard’s fragmentary narrative may encourage emotional introspection; however, are books’ philosophical musings on life and suffering more influential? If academics can identify their work’s strongest EI messages, they can assist teachers in selecting literary pieces in the classroom.

It may also be helpful to examine how writers and literary strategies affect EI. Does Quignard’s writing compare with that of other intellectually and emotionally difficult authors? Researchers may compare Quignard’s EI benefits with Jean-Paul Sartre and Albert Camus to determine whether they are genre-specific or based on literary themes. Through comparison, we can see how literature affects EI across cultures.

In addition, future research should include the cultural setting of the medicines. This study only evaluated a homogeneous sample of students, although students from diverse cultures may interact differently with literature. Similar studies with students from various cultures might help us determine whether Quignard’s work equally affects EI development across populations. Future research should use a more diverse sample and analyse generalisability to determine whether literature-based EI therapies work in various cultures.

Finally, while studying Quignard’s influence on students’ EQ, researchers can add various writers, topics, and tale styles. How do first- and third-person views affect student character relationships? Are love, redemption, and loss themes more likely to provoke feelings that help readers build EI? Better knowledge of how literature affects EI should inform future curriculum choices, and literary interventions should optimise emotional growth.

This study shows that Pascal Quignard’s novels may boost the EI of high school students. Quignard’s moving work can help students develop empathy, self-awareness, and emotional regulation. These findings should guide future studies on long-term literary treatment benefits and EuroQol-boosting strategies. Further study suggests that books, especially those with philosophical depth and emotional complexity, such as Quignard’s, can help youngsters develop and improve their emotions.

While this study focused on Pascal Quignard’s emotionally complex narratives, the underlying pedagogical model, pairing emotionally rich literature with structured EI reflection and measurement, can be applied across genres and disciplines. Educators might select culturally relevant novels, poetry, or even film scripts that evoke similar themes of identity, memory, or moral tension. By guiding students through targeted reflection activities and integrating EI assessments, teachers in diverse educational contexts can foster self-awareness, emotional regulation, and empathy, regardless of the specific author or medium.

## Data Availability

The raw data supporting the conclusions of this article will be made available by the authors, without undue reservation.
